# Outcomes of inferior vena cava filtration in lung transplant recipients

**DOI:** 10.3389/fradi.2026.1796398

**Published:** 2026-03-23

**Authors:** Kiyon Naser-Tavakolian, Abin Sajan, Venkatesh P. Krishnasamy, Tarub Mabud, John Filtes, Asad Baig, Debkumar Sarkar, Stephen P. Reis

**Affiliations:** 1 Vascular and Interventional Radiology, Columbia University, New York, NY, United States; 2 Vascular and Interventional Radiology, University of Alabama, Birmingham, AL, United States

**Keywords:** DVT, filter retrieval, inferior vena cava filters, lung transplantation, venous thromboembolism

## Abstract

**Objective:**

To evaluate the safety, retrieval outcomes, and procedural characteristics of retrievable inferior vena cava (IVC) filters placed in lung transplant recipients with venous thromboembolism.

**Methods:**

This retrospective single-center study included adult lung transplant recipients who underwent retrievable IVC filter placement between January 2021 and April 2025. Collected variables included patient demographics, indication for filter placement, filter type and position, anticoagulation status, retrieval technique, dwell time, fluoroscopy time, radiation dose, complications, and survival. Retrieval probability over time was assessed using Kaplan–Meier analysis.

**Results:**

Ninety-five lung transplant recipients (44 men, 51 women; mean age, 60.3 years) underwent retrievable IVC filter placement. Indications included deep vein thrombosis alone in 74 patients (77.9%), combined deep vein thrombosis and pulmonary embolism in 20 (21.1%), and isolated pulmonary embolism in one (1.1%). Filters included Option Elite (*n* = 93) and Celect (*n* = 2), with infrarenal placement in 89 patients (93.7%). Sixty-three filters (66.3%) were retrieved after a median dwell time of 7.7 months (interquartile range, 4–10). Retrieval techniques included snare (*n* = 53) and forceps-assisted (*n* = 10) with 100% technical success. Mean fluoroscopy time was 8.4 ± 6.5 min, and mean radiation dose was 234.3 ± 200.7 mGy. One filter-related complication (1.1%) occurred, consisting of filter migration into the right renal vein, which was successfully removed without injury; no filter-related deaths were observed.

**Conclusion:**

In this single-center retrospective cohort, retrievable IVC filters were managed in lung transplant recipients with a low complication rate and a majority undergoing retrieval during follow-up.

## Introduction

Lung transplantation, a life-saving treatment for end-stage lung disease, carries a substantial risk of venous thromboembolism (VTE), with reported incidences ranging from 6%–45% (1–3). This increased risk reflects a combination of factors, including immobility, surgical trauma, postoperative coagulopathy, and immunosuppressive therapy (4). Pulmonary embolism (PE) is particularly concerning in this population because of the absence of a bronchial collateral blood supply, which increases the risk of pulmonary infarction and associated mortality (5). Inferior vena cava (IVC) filters are frequently used in lung transplant recipients when anticoagulation must be interrupted—most commonly during bronchoscopic procedures or in the setting of bleeding complications (6, 7). While these devices offer temporary protection against PE, evidence regarding their safety, retrieval success, and long-term outcomes in lung transplant recipients remains limited. Studies in the general population report retrieval rates of 30% to 50% and complication rates of up to 10%, including filter migration, thrombosis, fracture, and perforation (1, 8–10). Data specific to solid organ transplant patients are sparse. In one small series, Dowell et al. reported a retrieval rate of only 6% among transplant recipients, including two lung transplant patients (11).

Because lung transplant patients often experience prolonged hospitalization, repeated invasive procedures, and fluctuating anticoagulation needs, they represent a unique subgroup in whom IVC filter use may be more common. This study was undertaken to evaluate the safety, retrieval rates, and clinical outcomes of IVC filter placement in lung transplant recipients. We hypothesized that, with structured follow-up and multidisciplinary management, filters could be retrieved safely and at higher rates than previously reported.

## Methods

This IRB-approved, retrospective cohort study included all lung transplant recipients who underwent retrievable IVC filter placement at a single tertiary care center between January 2021 and April 2025. Eligible patients were adults (≥18 years) who received the filter post-transplantation for VTE-related indications, including deep vein thrombosis (DVT), pulmonary embolism (PE) or both, or due to temporary contraindications to anticoagulation. Exclusion criteria comprised pediatric patients (<18 years), non-lung transplant recipients, patients with filters placed prior to transplantation, those receiving permanent (non-retrievable) filters, and filters placed at outside institutions.

Clinical data were extracted from electronic medical records and included demographic characteristics (age, sex), transplant date, VTE features (type, location, timing), filter details (type, date, and position), anticoagulation status, retrieval outcomes (technique, fluoroscopy time, radiation dose), adverse events, and survival. Adverse events were classified using the Society of Interventional Radiology (SIR) adverse event classification system (12). Descriptive statistics were used to summarize patient and procedural characteristics. Continuous variables were reported as means (standard deviation, SD) or medians (interquartile range, IQR), and categorical variables as frequencies and percentages. Differences between retrieval techniques were assessed using standard comparative statistics, with statistical significance defined as *p* < 0.05. Kaplan–Meier analysis was used to estimate the probability of filter retrieval over time, with censoring for non-retrieved filters at study end or at death.

All filters were placed under fluoroscopic guidance via the internal jugular or common femoral vein. Patients were scheduled for follow-up in the interventional radiology clinic 1–3 months after placement to reassess the need for continued filtration. Multidisciplinary review with pulmonology, cardiothoracic surgery, and transplant medicine was performed before scheduling retrieval. Filters removed within 90 days were typically managed in an office-based laboratory, whereas those with longer dwelling times were removed as outpatient procedures at the hospital.

## Results

A total of 95 lung transplant recipients [51 women (55.8%); mean age, 60.3 ± 12.5 years] underwent IVC filter placement during the study period. The primary indication was deep vein thrombosis (DVT) alone in 74 patients (77.9%), followed by combined DVT and pulmonary embolism (PE) in 20 (21.1%) and isolated PE in one (1.1%). The most common sites of DVT were the common femoral vein (*n* = 72) and the popliteal vein (*n* = 26).

Most filters were Option Elite devices (*n* = 93, 97.9%) (Argon, Plano, TX), with two Celect filters (2.1%) (Cook Medical, Bloomington, IN). Eighty-nine filters (93.7%) were placed in the infrarenal position, and six (6.3%) were placed suprarenally because of IVC thrombus. At the time of VTE diagnosis, patients were started on continuous heparin infusion and later transitioned to a direct oral anticoagulant or low-molecular-weight heparin in most cases [apixaban, *n* = 79 [83.2%]; enoxaparin, *n* = 8 [8.4%]; heparin, *n* = 6 [6.3%]]. Two patients remained off anticoagulation because of bleeding, and the six patients maintained on heparin were still hospitalized at the time of data collection.

Of the 95 filters placed, 63 (66.3%) were successfully retrieved, with a median dwell time of 7.7 months (IQR, 4–10). Retrieval was performed using a snare technique in 51 cases (81.0%), forceps in 10 (15.9%), and combined hangman-plus-snare in two (3.2%). All retrieval attempts were technically successful. The mean fluoroscopy time was 8.4 ± 6.5 min, with forceps retrieval requiring significantly longer time than snare retrieval (14.3 ± 8.0 min vs. 7.0 ± 5.6 min; *p* = 0.0006). Mean radiation dose was 234.3 ± 200.7 mGy, higher for forceps retrievals (356.3 ± 247.3 mGy) than for snare (204.6 ± 183.0 mGy; *p* = 0.0268). These findings are summarized in Table 1.

**Table 1:** Summary of IVC filter outcomes in lung transplant recipients.

Variable	Value
Total Patients, *n*	95
Male, Female *n* (%)	44 (44.2), 51 (55.8)
Mean Age, years (SD)	60.3 (12.5)
Indication For Filter Placement:	
DVT Only, *n* (%)	74 (77.9)
DVT + PE, *n* (%)	20 (21.1)
PE Only, *n* (%)	1 (1.1)
Infrarenal Filter, *n* (%)	89 (93.7)
Suprarenal Filter, *n* (%)	6 (6.3)
Filters Retrieved, *n* (%)	63 (66.3)
Median dwell time, months (IQR)	7.7 (4–10)
Retrieval technique, *n* (%)	
Snare	53 (84.1)
Forceps	10 (15.9)
Mean Fluoroscopy Time, min (SD)	8.4 (6.5)
Mean Radiation Dose, mGy (SD)	234.3 (200.7)
Adverse Events, *n* (%)	1/95 (1.1)
Filter Type, *n* (%)	
Option Elite	93 (97.9)

SD, standard deviation; DVT, deep vein thrombosis; PE, pulmonary embolus.

**Figure 1:**
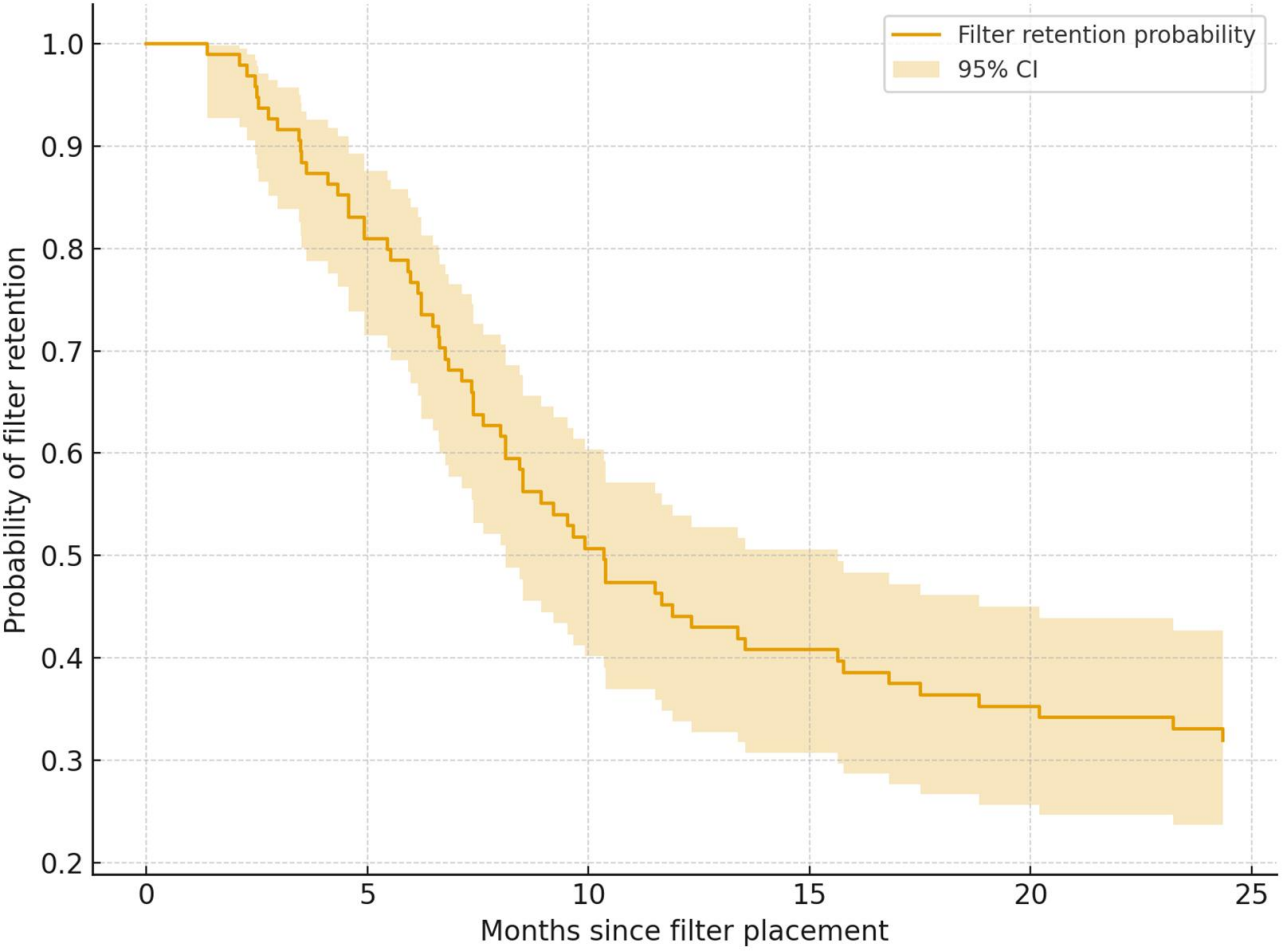
Kaplan–Meier curve demonstrating inferior vena cava (IVC) filter retention probability over time. The *y*-axis represents the estimated probability of filter retention, and the *x*-axis represents time from filter placement. Shaded bands indicate the 95% confidence interval. Patients were censored at the time of filter retrieval or last clinical follow-up.

Kaplan–Meier analysis anchored to the date of filter placement demonstrated a rapid decline in filter retention probability within the first year following implantation. The probability of filter retention was approximately 51% at 10 months (95% CI per table), indicating a nearly 50% decrease in retention relative to baseline. Retention further decreased to approximately 29% by the end of follow-up (approximately 50 months). Most retrievals occurred within the first 10 months, consistent with active surveillance and timely filter management in the lung transplant cohort (Figure 1). One (1.1%) mild adverse event (SIR Classification AE Severity 1), consisting of filter migration into the right renal vein occurred. The filter was successfully retrieved with forceps, and no filter-related deaths were observed. Non-retrieved filters (*n* = 32, 33.7%) were most often retained due to persistent VTE (*n* = 7), ongoing hospitalization or repeated bronchoscopic procedures (*n* = 15), episodes of bleeding from anticoagulation thus requiring the filter to remain in place (*n* = 2), unrelated death (*n* = 4), or loss to follow-up (*n* = 2). In a few cases, filters were not retrieved because of recent placement (<3 months from study end) (*n* = 2). No IVC filter related deaths were observed in this cohort.

## Discussion

IVC filter placement remains an important consideration in lung transplant recipients who develop DVT or PE early after transplantation, particularly when consistent anticoagulation is not feasible in the immediate postoperative period. This study demonstrates that IVC filters can be used safely and effectively in this population, achieving a 66.3% retrieval rate, which is substantially higher than the 30%–50% typically reported in the general population and markedly higher than the 6% rate previously reported among solid organ transplant recipients by Dowell et al. (8, 9, 11). In that series, only two patients had undergone lung transplantation, further emphasizing the scarcity of data specific to this group.

Our results also show that most retrievals occurred within 10 months, as reflected in the Kaplan–Meier analysis, suggesting that structured surveillance programs are effective in facilitating timely removal. The low complication rate (1.1%) aligns with studies reporting filter migration in <2% of cases, with successful management using advanced retrieval techniques (13, 14). No filter thrombosis, filter fracture, PE, or perforation were noted. The small subset of suprarenal filter placements (6.3%), performed in cases of IVC thrombus, highlights procedural flexibility when anatomy or thrombus burden precludes standard positioning (15). The predominance of apixaban use (83.2%) reflects current practice trends favoring direct oral anticoagulants for VTE management, though its advantage in transplant recipients remains unproven (16, 17). The longer fluoroscopy times and higher radiation exposure observed with forceps retrieval likely reflect their use following failed snare attempts. Non-retrieval was primarily driven by persistent VTE or ongoing bronchoscopic procedures, emphasizing the need for tailored filter management (11). IVC filter related mortality was 0% but overall mortality in this cohort was 4.2%, concordant with prior evaluations of lung transplant patients albeit those with VTE (18). These findings underscore the importance of structured follow up to optimize retrieval timing and minimize complications (19).

Current Society of Interventional Radiology (SIR) guidelines, last updated in 2020, recommend that inferior vena cava (IVC) filter placement be reserved for patients with acute VTE who have an absolute contraindication to anticoagulation, or for those with recurrent pulmonary embolism (PE) despite adequate anticoagulation (20). The guidelines recommend retrieval of retrievable filters as soon as clinically appropriate once the risk of PE has been mitigated, and stress the importance of structured follow-up programs to ensure timely removal and minimize filter-related complications (19, 20). Separately, the U.S. Food and Drug Administration (FDA), in its 2014 Safety Communication, cited a decision analysis suggesting that the risk-benefit profile favors filter removal between 29 and 54 days after implantation once the transient risk of PE has resolved. Routine placement for primary prophylaxis is discouraged due to the lack of demonstrated mortality benefit and the increased risk of filter-related thrombosis, migration, or fracture (21).

In this context, lung transplant recipients represent a unique population not specifically addressed in existing guidelines. The SIR recommendations were derived primarily from studies in general, trauma, or oncology populations, with minimal data on solid organ transplant cohorts. Early after lung transplantation, patients often face temporary contraindications to anticoagulation due to bleeding risk, airway anastomotic healing, recurrent bronchoscopic procedures, or hemodynamic instability. These clinical circumstances fall within a gray zone of temporary contraindication, where current SIR recommendations provide little transplant-specific guidance.

The median dwell time of 7.7 months in our cohort substantially exceeds the 29- to 54-day window cited by the FDA and likewise extends beyond the SIR's general recommendation to retrieve filters as soon as clinically appropriate. However, this discrepancy reflects the clinical reality of this population rather than failure of the retrieval program. Lung transplant recipients face recurrent and often overlapping contraindications to anticoagulation throughout their post-transplant course, including scheduled bronchoscopic surveillance, airway anastomotic complications, infectious events, and episodic bleeding, which collectively delay both anticoagulation initiation and filter retrieval in an iterative, individualized fashion. Critically, despite the prolonged implantation duration, only one filter-related adverse event (1.1%) was observed, and no filter-related deaths occurred, suggesting that extended dwell time, when managed within a structured multidisciplinary surveillance program, does not necessarily translate to increased clinical harm in this population. These findings suggest that individualized retrieval timing linked to transplant-specific clinical milestones, rather than adherence to fixed intervals derived from general populations, may be both safe and appropriate in lung transplant recipients.

The findings of this study, a 66.3% retrieval rate and 1.1% complication rate, demonstrate that IVC filters can be safely and effectively managed in the lung transplant population under a structured, multidisciplinary follow-up protocol. Our institution will coordinate a follow up appointment within 3 months after IVC filter placement on the day the filter is placed. The patient is then reassessed every 3 months with discussion with the transplant team and pulmonology regarding possible IVC filter removal. Criteria for IVC filter removal was met when the patient could be on continuous anticoagulation without interruptions for transplant related procedures or had completed their anticoagulation course and had a negative venous duplex of the lower extremities at the time of follow up. The retrieval rate exceeds those reported in general populations and suggests that close coordination between transplant and interventional radiology teams can overcome one of the major challenges cited by SIR: poor adherence to retrieval follow-up.

This experience supports consideration of clarifying transplant specific indications for temporary IVC filter placement in select lung transplant recipients. Potential applications include the early post-transplant period when hemodynamic instability or surgical bleeding precludes anticoagulation, during recurrent bronchoscopic interventions requiring intermittent cessation of anticoagulants, and in perioperative contexts such as reoperation, airway complications, or extracorporeal membrane oxygenation (ECMO) decannulation. Filters may also serve as a bridge when anticoagulation cannot be promptly resumed after procedural or infectious complications.

Retrieval criteria could likewise benefit from proactive, standardized timing protocols linked to transplant follow-up schedules, for example, evaluating filter status within 3 to 6 months post-placement in coordination with routine bronchoscopy assessments. These structured timepoints could act as “safety checkpoints,” reducing long-term dwell times and minimizing complications (19, 20). By contributing outcome data specific to this complex population, this study adds to the evidence base that could inform future revisions of SIR guidelines, particularly in defining temporary indications and structured surveillance standards for high-risk surgical patients. Multicenter, prospective studies are warranted to validate these findings, refine optimal retrieval intervals, and better define the risk–benefit profile of IVC filter use in solid organ transplant recipients.

This study has several limitations. It is retrospective and reflects the experience of a single high-volume transplant center using predominantly one filter type, which limits generalizability. Some patients lacked complete follow-up, potentially leading to underestimation of complications. Retrieval rates may also appear higher in this structured environment compared with centers lacking dedicated surveillance programs. The absence of a control group such as non-transplant recipients or transplant patients managed without IVC filters limits comparative interpretation. Because this study was limited to patients who underwent IVC filter placement, it does not capture lung transplant recipients with VTE who were managed exclusively with anticoagulation and therefore does not reflect the overall incidence of VTE in the transplant population. Additionally, the sample size was insufficient to support a robust multivariable analysis of predictors of non-retrieval or delayed retrieval; such an analysis in this cohort would risk overfitting and unreliable estimates. This question is an important area for future investigation in larger, multicenter cohorts.

Long-term follow-up beyond retrieval was incomplete, so delayed complications such as IVC stenosis, recurrent thrombosis, or filter migration may have been underreported. Retrieval timing was individualized rather than protocolized, introducing variability in dwell time and outcomes. Confounding factors typical of post-transplant recovery, including infection, airway complications, and prolonged hospitalization may also have affected retrieval feasibility independent of filter performance. Finally, cost-effectiveness and workflow differences between inpatient and outpatient retrieval were not evaluated but could inform future program development.

## Conclusion

IVC filters can be safely removed at high retrieval rates with minimal complications in lung transplant recipients. These findings highlight that filters can be managed effectively even in complex post-transplant populations with structured surveillance and multidisciplinary coordination. Incorporating proactive follow-up protocols and individualized risk–benefit assessments is critical to optimizing outcomes and minimizing long-term complications. Moreover, the high retrieval success observed in this cohort suggests that similar structured approaches could be adopted to inform future updates to the Society of Interventional Radiology (SIR) guidelines, particularly regarding temporary indications and standardized retrieval timing in high-risk surgical patients.

## Data Availability

The raw data supporting the conclusions of this article will be made available by the authors, without undue reservation.
